# Miliary pulmonary nodules due to *Mycobacterium xenopi* in a steroid-induced immunocompromised patient successfully treated with chemotherapy: a case report

**DOI:** 10.1186/s12890-016-0252-y

**Published:** 2016-06-10

**Authors:** Yoshio Okano, Tsutomu Shinohara, Shino Imanishi, Naoki Takahashi, Nobuhito Naito, Takanari Taoka, Naoki Kadota, Fumitaka Ogushi

**Affiliations:** Division of Pulmonary Medicine, National Hospital Organization Kochi Hospital, 1-2-25 Asakuranishimachi, Kochi, 780-8077 Japan; Department of Clinical Investigation, National Hospital Organization Kochi Hospital, 1-2-25 Asakuranishimachi, Kochi, 780-8077 Japan

**Keywords:** *Mycobacterium xenopi*, Miliary nodules, Immunocompromised patient

## Abstract

**Background:**

*Mycobacterium xenopi*-infected patients have a high prevalence of pulmonary cavities and nodules. However, the clinical course for patients with miliary nodules due to *M. xenopi* has not yet been reported.

**Case presentation:**

We encountered a case of miliary nodules with gradually worsening coughing and sputum production in a 44-year-old male who had renal dysfunction due to glomerulosclerosis with a decade-long history of steroid therapy. Although we started anti-tuberculosis treatment on clinical suspicion of miliary tuberculosis, cultures of sputum and bronchial lavage were both positive for *M. xenopi*. The patient was successfully treated with rifampin, ethambutol and clarithromycin, without fibrosis. It was unclear whether the miliary pattern was induced by hematogenous or endobronchial spread of the *M. xenopi* infection.

**Conclusion:**

Even when clinical and radiological disease manifestations are similar to those of miliary tuberculosis, *M. xenopi* infection should be considered in the differential diagnosis of miliary nodules.

## Background

*Mycobacterium xenopi* is one of the common causes of nontuberculous mycobacteria (NTM) pulmonary disease, especially in Canada, France and the United Kingdom [1–3]. In comparison with *M. avium* complex (MAC), *M. xenopi*-infected patients were found to have a high prevalence of pulmonary cavities and nodules, and rarely present a nodular bronchiectatic form [4]. A large number of patients with pulmonary cavities due to *M. xenopi* simultaneously had computed tomography (CT) findings of random nodules or consolidation rather than the fibrocavitary form [4]. The management of individual patients with *M. xenopi* is difficult, since a standard treatment has not been established.

Regarding the random nodules, 4 cases of miliary nodules due to *M. xenopi* with acquired immunodeficiency syndrome were reported by Bankier et al. However, the radiological evaluation of these cases was made by chest X-ray, not by CT. In addition, the clinical course for the patients was not described [5]. In this report, we present a case of chest CT-proven miliary nodules due to *M. xenopi* without cavities and consolidations in a steroid-induced immunocompromised patient successfully treated with chemotherapy.

## Case presentation

**Fig. 1:**
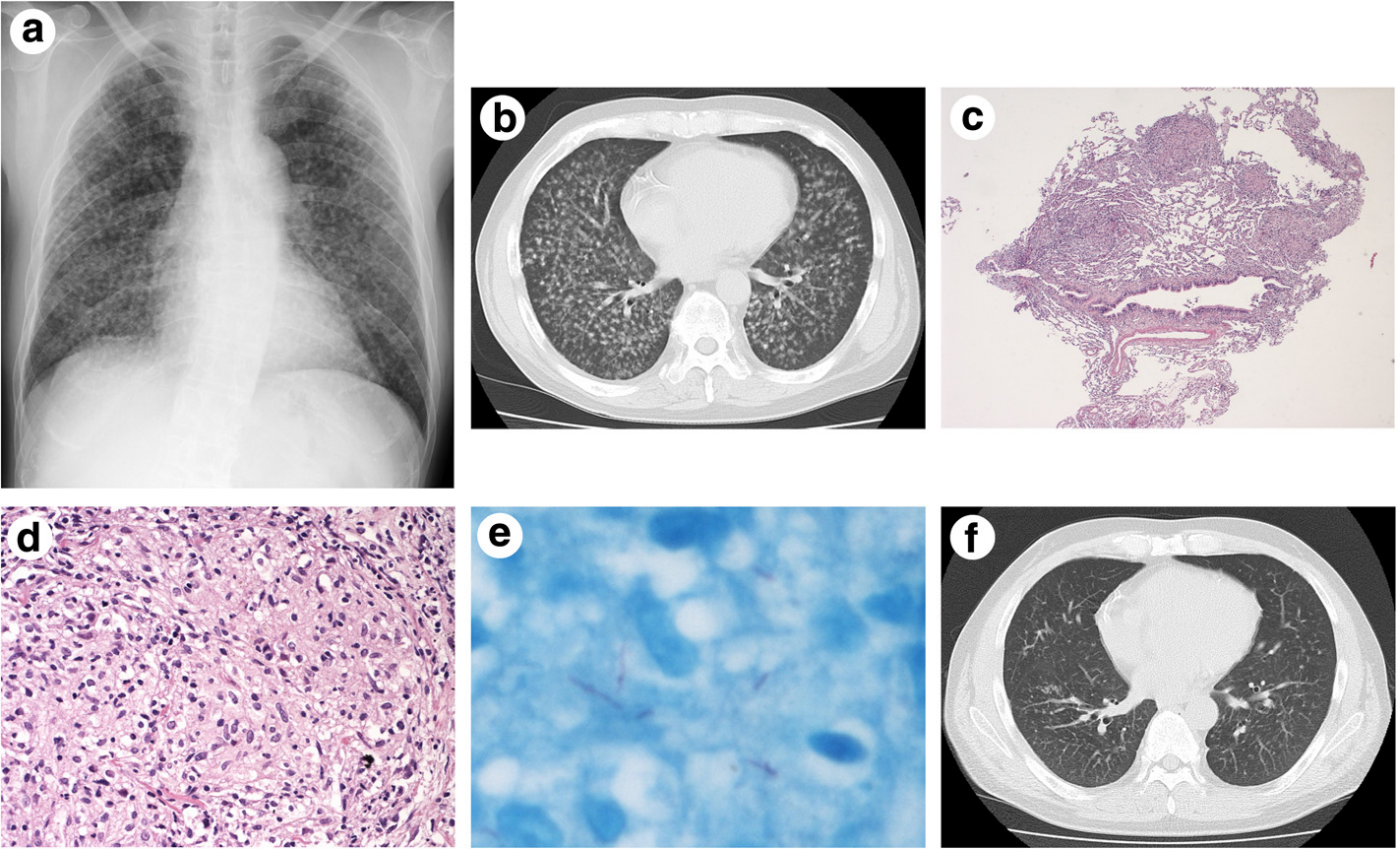
Chest X-ray (**a**), CT (**b** and **f**) and pathological findings of transbronchial lung biopsy (**c** and **d**, hematoxylin and eosin staining; **e**, Ziehl-Neelsen staining). **a** and **b**: A high number of miliary nodules in both lungs without consolidations and cavities at presentation. **c** and **d**: Granulomatous inflammation (**c**, low-power field; **d**, high-power field). **e**: Bacillus bodies seen as a bright red rod. **f**: Marked improvement in miliary nodules after 15 months of chemotherapy

A 44-year-old male, who had renal dysfunction due to glomerulosclerosis with a decade-long history of steroid therapy (10–20 mg of prednisone per day), visited our department because of gradually worsening coughing and sputum production. He had no past history of any pulmonary disease, such as chronic obstructive pulmonary disease (COPD), tuberculosis or bronchial asthma. The results of a physical examination were normal. Laboratory data were as follows: white blood cell count, 10630/μl (neutrophils, 91.6 %); hemoglobin, 12.5 g/dl; platelet count, 61.2 x 10^4^/µl; C-reactive protein, 6.27 mg/dl; creatinine, 1.64 mg/dl. Chest X-ray and CT showed a high number of miliary nodules in both lungs without consolidations or cavities (Fig. 1a and b). Underlying pulmonary diseases, such as emphysema and bronchiectasis, were not observed. Abdominal CT and magnetic resonance imaging of the spine detected no extrapulmonary lesions. Although a sputum acid-fast bacillus smear was positive, polymerase chain reaction (PCR) tests for *M. tuberculosis*, *M. avium* and *M. intracellurare* of the sputum were all negative. On clinical suspicion of miliary tuberculosis, we started quadruple therapy (isoniazid, rifampin (RFP), ethambutol (EB) and pyrazinamide) without dose reduction of prednisone (10 mg/day). Then, we carried out a bronchoscopic examination, and transbronchial lung biopsy detected small granuloma (Fig. 1c and d) and Ziehl-Neelsen staining-positive bacillus bodies (Fig. 1e). Acid-fast bacillus smear and PCR tests of bronchial lavage showed the same results as sputum. Later, the culture of sputum and bronchial lavage obtained mycobacterium colonies, and the isolates from both samples were identified as *M. xenopi* by DNA-DNA hybridization (DDH). Since patients infected with *M. heckeshornense* may have been included in the cases that were diagnosed as *M. xenopi* disease by DDH, we reconfirmed that the isolate from bronchial lavage was *M. xenopi* by sequence analyses of the 16S rRNA gene, *rpoB* and *hsp65* [6]. After the final diagnosis of *M. xenopi* pulmonary infection was established, we changed the quadruple therapy to treatment with RFP (450 mg/day), EB (750 mg/day) and clarithromycin (CAM) (800 mg/day). Chemotherapy was continued for a total period of 15 months without side effects, and steroid therapy for glomerulosclerosis was also continued without dose modification. Sputum cultures yielded no mycobacteria in the last 12 months. After the chemotherapy, chest CT showed a prominent reduction in the size and number of miliary nodules without fibrosis (Fig. 1f).

## Discussion

Clinical and radiological disease manifestations of *M. xenopi* infection vary according to each person’s immunological status, and can be classified into three groups: (1) a cavitary form in patients with pre-existing pulmonary disease; (2) a solitary nodular form in immunocompetent patients and (3) an acute infiltrate form in immunosuppressed patients [2]. Although multiple small nodules (<5 mm) coexisting with a predominant form were a common finding in patients with *M. xenopi* pulmonary infection, the mean number is about 10–16 per patient [4, 7]. Therefore, the CT-proven miliary pattern without cavities and consolidations observed in our immunocompromised patient could be a distinct entity.

Previously, O’Connell et al. reported an autopsy-based series of pulmonary or disseminated nontuberculous mycobacterial disease. In that report, a miliary pattern was noted in 2 of the 5 disseminated patients with mycobacterial lung involvement, but not in the 11 primary pulmonary mycobacterial diseases without extrapulmonary infection [8]. In addition, miliary pulmonary infection with a macropapular rash on extremities caused by *M. terrae* in an immunocompromised patient was reported [9]. Miliary pulmonary infection due to nontuberculous mycobacteria in a patient without extrapulmonary infections may be extremely rare.

Although a miliary pattern is usually induced by the hematogenous spread of infection or tumor cells, this pattern with or without ground-glass opacities sometimes can be seen in so-called hot tub lung (HTL) caused by inhalation of water aerosol containing NTM, especially the MAC [10–12]. There seems to be quite a controversy over whether HTL is a direct appearance of mycobacterial infection or is a hypersensitivity pneumonitis (HP). Because the sputum cultures continued to be positive for 6 weeks after initiation of chemotherapy, an infectious process was probably involved in the pathogenesis in our case. While acid-fast bacillus cultures of sputum, tissue and bronchial lavage from patients with HTL are positive at high rates, HTL usually manifests spontaneous or steroid-induced improvement after cessation of hot tub exposure without antimycobacterial treatment [12]. It was also reported in a mouse model that HTL develops through mycobacterial engagement with Toll-like receptor 9/Myeloid differentiation factor 88 signaling in pulmonary CD11b-positive dendritic cells, regardless of the mycobacterial infectious capacity [13]. In addition, intravesical administration of bacillus Calmette-Guerin (BCG) against urothelial cancer occasionally induces systemic hypersensitivity reactions, including pneumonitis, without detection of BCG in corresponding organs [14]. These data indicate that NTMs have pathogenic potential not only as infectious microorganisms, but also as highly immunogenic substances for HP. Although HTL generally appears in immunocompetent patients with no preexisting lung disease [12], the hypersensitivity phenomenon may have overlapped, to a greater or lesser degree, with an infectious process in the pathogenesis in our case. From the above-mentioned points, the possibility remains that the miliary pattern of our patient resulted from endobronchial spread of the *M. xenopi* infection with hypersensitivity to *M. xenopi* antigens.

Although a standard treatment for *M. xenopi* has not been established, ATS/IDSA recommends an RFP, EB and CAM-containing regimen [15]. In general, the prognosis for the hematogenous spread of infection in immunocompromised patients is poor, but a prognosis for NTM miliary infection has not been well documented. In this case, treatment with RFP, EB and CAM was effective for miliary pulmonary infection caused by *M. xenopi* even in an immunocompromised patient. Further clinical studies on the pathophysiology related to *M. xenopi* miliary infection are needed in order to improve the management of this rare condition.

## Conclusion

*M. xenopi* infection should be considered in the differential diagnosis of miliary pulmonary nodules, even when the clinical and radiological disease manifestations are similar to those of miliary tuberculosis.

## Abbreviations

NTM, nontuberculous mycobacteria; *M. xenopi*, *Mycobacterium xenopi*; MAC, *M. avium* complex; CT, computed tomography; COPD, chronic obstructive pulmonary disease; PCR, polymerase chain reaction; RFP, rifampin; EB, ethambutol; DDH, DNA-DNA hybridization; CAM, clarithromycin; HTL, hot tub lung; HP, hypersensitivity pneumonitis; BCG, bacillus Calmette-Guerin
